# Comparative Analysis of Gastric Epithelial Neoplasm of Fundic‐Gland Mucosa Lineage: Histopathological Features of Background Gastric Mucosa

**DOI:** 10.1002/jgh3.70324

**Published:** 2025-12-07

**Authors:** Ryo Watanabe, Tomoyuki Yada, Takashi Oide, Miki Yoshinobu, Yugo Kawasaki, Masaaki Mino, Keita Odaka, Katsunori Sekine, Naomi Uemura

**Affiliations:** ^1^ Department of Gastroenterology and Hepatology National Kohnodai Medical Center, Japan Institute for Health Security Chiba Japan; ^2^ Department of Pathology and Laboratory Medicine National Kohnodai Medical Center, Japan Institute for Health Security Chiba Japan

**Keywords:** Fundic glands, gastric adenocarcinoma, gastric mucosa, *helicobacter* infections, stomach neoplasms

## Abstract

**Aims:**

Gastric epithelial neoplasm of fundic‐gland mucosa lineage (GEN‐FGML) has been increasingly recognized in recent years; however, few studies have investigated the histopathology of the background gastric mucosa surrounding the lesion. This study clarifies the histopathological features of the background gastric mucosa in GEN‐FGML.

**Methods and Results:**

A retrospective analysis was conducted of 30 GEN‐FGML lesions (28 patients) diagnosed at our institution between December 2012 and 2023, excluding cases of gastric adenocarcinoma of fundic‐gland mucosa type. Patients were classified according to *Helicobacter pylori* infection status, and clinicopathological features were compared. The background gastric mucosa was evaluated using the Updated Sydney System (USS). In total, 15 lesions (13 patients) were in the uninfected group, and 15 lesions (15 patients) were in the past *H. pylori* infection group (i.e., the infected group); no lesions from patients with current infection were included. In the uninfected group, none of the lesions showed histopathological atrophy or intestinal metaplasia. Conversely, histopathological atrophy was observed in 12 lesions in the infected group. Although 86.7% (13/15 lesions) of the infected cases were endoscopically located in nonatrophic areas, 10 displayed mild histopathological atrophy (USS 1+).

**Conclusion:**

Oxyntic gland adenoma and gastric adenocarcinoma of fundic‐gland type arise predominantly from mildly atrophic mucosa with preserved fundic glands in previously infected stomachs, and, albeit less frequently, from severely atrophic mucosa. During routine endoscopic examinations, careful observation of the fundic gland is warranted regardless of the presence of background mucosal atrophy.

## 1 Introduction

Gastric adenocarcinoma showing differentiation toward fundic gland cells was first reported by Tsukamoto et al. in 2007 as “gastric adenocarcinoma with chief cell differentiation” [1]. In 2010, Ueyama et al. reclassified it as “gastric adenocarcinoma of fundic‐gland type (chief cell predominant type)” [2]. In the 5th edition of the WHO Classification of Tumors (2019), intramucosal lesions were designated as “oxyntic gland adenoma” (OGA) and submucosal invasive lesions as “gastric adenocarcinoma of fundic‐gland type” (GA‐FG), introducing these as new pathological entities [3]. In 2021, Ueyama et al. proposed the term “gastric epithelial neoplasm of fundic‐gland mucosa lineage” (GEN‐FGML) as a pathological category encompassing OGA, GA‐FG, and gastric adenocarcinoma of fundic‐gland mucosa type (GA‐FGM) [4].

GEN‐FGML was initially thought to arise in nonatrophic gastric mucosa without *Helicobacter pylori* infection; however, in recent years, it has also been reported in cases with current or past *H. pylori* infection. In cases of GEN‐FGML arising in atrophic mucosa, it remains unclear whether the neoplasm develops only in areas with abundant preserved fundic glands, or also in regions with reduced or minimal fundic gland presence. Although the clinicopathological features of GEN‐FGML have been progressively clarified, few studies have examined in detail the histopathological characteristics of the background gastric mucosa surrounding the lesion. Therefore, the aim of this study was to clarify the histopathological features of the background gastric mucosa in GEN‐FGML.

## 2 Methods

### 2.1 Patients

This single‐center, retrospective study included 30 GEN‐FGML lesions (28 patients) diagnosed at our institution between December 2012 and 2023. Cases of GA‐FGM were excluded. One patient who was diagnosed with GEN‐FGML using endoscopic biopsy but in whom no neoplastic lesion was identified in the resected specimen was excluded. Definitive diagnoses were made using specimens obtained by endoscopic submucosal dissection (ESD). Hematoxylin and eosin staining as well as immunohistochemical staining for pepsinogen I (7G3, Abcam; 1:100), MUC6 (CLH5, Novocastra; 1:100), MUC5AC (CLH2, Novocastra; 1:200), CD10 (56C6, Novocastra; 1:400), and MUC2 (Ccp58, Novocastra; 1:200), were performed, and the findings were evaluated by a single pathologist.

This study was approved by the Ethics Committee of the National Center for Global Health and Medicine (NCGM‐S‐004421‐01) and conducted in accordance with the ethical principles of the Declaration of Helsinki.

### 2.2 Endoscopic and Histopathological Evaluation of the Gastric Mucosa

Endoscopic evaluations were performed using EVIS LUCERA ELITE or EVIS X1 with high‐resolution endoscopes (GIF‐H290 series and GIF‐XZ1200, Olympus Medical Systems, Tokyo, Japan). Gastric mucosal atrophy was classified endoscopically according to the Kimura–Takemoto classification [5] into the following categories: no atrophy (C0), mild atrophy (C1–C2), moderate atrophy (C3–O1), and severe atrophy (O2–O3). The endoscopic atrophy was assessed based on this classification by two expert endoscopists.

The background gastric mucosa surrounding the lesion was histopathologically evaluated in the non‐neoplastic mucosa at the lesion's largest cross‐section. Atrophy and intestinal metaplasia were assessed semi‐quantitatively based on the visual analogue scale of the Updated Sydney System (USS) [6], using a four‐grade scale: normal (−), mild (1+), moderate (2+), and marked (3+). Atrophy was evaluated to determine whether GEN‐FGML occurs in atrophic mucosa, regardless of the degree of residual fundic glands. Given that intestinal metaplasia often progresses alongside atrophy and is a well‐established risk factor for gastric cancer, it was also included and assessed using the four‐grade scale. Submucosal invasion was categorized according to the Japanese Classification, that is, SM1 (invasion < 500 μm from the muscularis mucosae) and SM2 (invasion ≥ 500 μm), with SM2 defined as deep submucosal invasion for international clarity.

### 2.3 Evaluation of *H. pylori* Infection Status

*H. pylori* infection status was determined based on endoscopic evaluations of gastric mucosal atrophy, a history of eradication therapy established through interviews, and results from at least one of the following tests: serological *H. pylori* IgG test (Eiken Chemical Co. Ltd. or Denka Co. Ltd., Tokyo, Japan; cut‐off value ≥ 10 U/mL considered positive), urea breath test (Otsuka Pharmaceutical Co. Ltd., Tokyo, Japan), *H. pylori* stool antigen test (Wakamoto Pharmaceutical Co. Ltd., Tokyo, Japan), or rapid urease test (Otsuka Pharmaceutical Co. Ltd.).

Current *H. pylori* infection: no history of eradication, with positive results in at least one diagnostic test and endoscopic evidence of gastric mucosal atrophy.

*H. pylori* ‐uninfected: no history of eradication, with negative results in at least one diagnostic test and no endoscopic evidence of gastric mucosal atrophy.

Past *H. pylori* infection: negative results in at least one diagnostic test, with a history of eradication and/or endoscopic evidence of gastric mucosal atrophy.

Cases with current or past *H. pylori* infection were classified as “infected,” whereas those without *H. pylori* infection were classified as “uninfected.”

### 2.4 Analysis Method

For the primary endpoint, the histopathological features of the background gastric mucosa surrounding the GEN‐FGML and other clinicopathological features were compared between the “infected” and “uninfected” groups.

### 2.5 Statistical Analysis

Statistical analyses were performed using EZR (version 1.55; Saitama Medical Center, Jichi Medical University, Saitama, Japan), a graphical user interface for R (The R Foundation for Statistical Computing, Vienna, Austria) [7]. Data were analyzed using Fisher's exact test and the Mann–Whitney U test, with significance set at *p* < 0.05.

## 3 Results

In total, 30 lesions (28 cases) were diagnosed as GEN‐FGML at our hospital, and their clinicopathological findings were analyzed (Table 1). The median age of the patients was 76 years, and there were more males (male–female ratio, 17:11). Regarding *H. pylori* infection status, 15 lesions (13 cases) were classified as the uninfected group and 15 lesions (15 cases) as past infection. Lesions from patients with current infection were excluded. All patients underwent ESD as the initial treatment.

**TABLE 1:** Clinicopathological features of GEN‐FGML.

Patients (*n* = 28)	
Sex (male/female)	17/11
Age (median)	76 [59–83]
Degree of endoscopic gastric mucosal atrophy (C0/C1–C2/C3–O1/O2–O3)	13/2/8/5
*Helicobacter pylori* infection (uninfected/past infection/current infection)	13/15
Multiple lesions (−/+)	27/1
Initial treatment (ESD/surgical resection)	28/0
Curability eCura (A/B/C‐1/C‐2)	12/13/0/3

Lesions (*n* = 30)	
Location (U/M/L)	22/8/0
Macroscopic type (elevated/flat/depressed)	13/10/7
Tumor size (mm, median [range])	5 [2–10]
Histological diagnosis (OGA/GA‐FG)	12/18
Depth [T1a(M)/T1b(SM1)/T1b(SM2)]	12/16/2
Depth of invasion (μm, median [range], *n* = 18)	113 [11–1250]
Lymphatic and/or venous invasion (−/+)	29/1
Horizontal margin (−/+)	30/0
Vertical margin (−/+)	30/0

Abbreviations: GA‐FG, gastric adenocarcinoma of fundic‐gland type; GEN‐FGML, gastric epithelial neoplasm of fundic‐gland mucosa lineage; OGA, oxyntic gland adenoma.

Lesions were predominantly located in the upper (U) third of the stomach, whereas none arose in the lower (L) third. Macroscopically, 13 lesions were classified as elevated type (0‐IIa), 10 as flat type (0‐IIb), and 7 as depressed type (0‐IIc), indicating that flat or depressed types accounted for 56.7% of all lesions. The median tumor size was 5 mm (range, 2–10 mm). Submucosal invasion was observed in 18 lesions (60.0%), comprising 16 SM1 and 2 SM2 lesions. All lesions were negative for horizontal and vertical margins; however, venous invasion was observed in one lesion. Pathological diagnoses included 12 OGA and 18 GA‐FG.

According to the Japanese Gastric Cancer Treatment Guidelines (6th edition) [8], curability classifications were eCura A in 12 cases, eCura B in 13 cases, and eCura C‐2 in 3 cases. All three patients with noncurative resections were followed up without additional treatment. The median follow‐up period was 35 months (range, 17–94 months), and no local recurrence or distant metastasis was observed during the follow‐up period.

**FIGURE 1:**
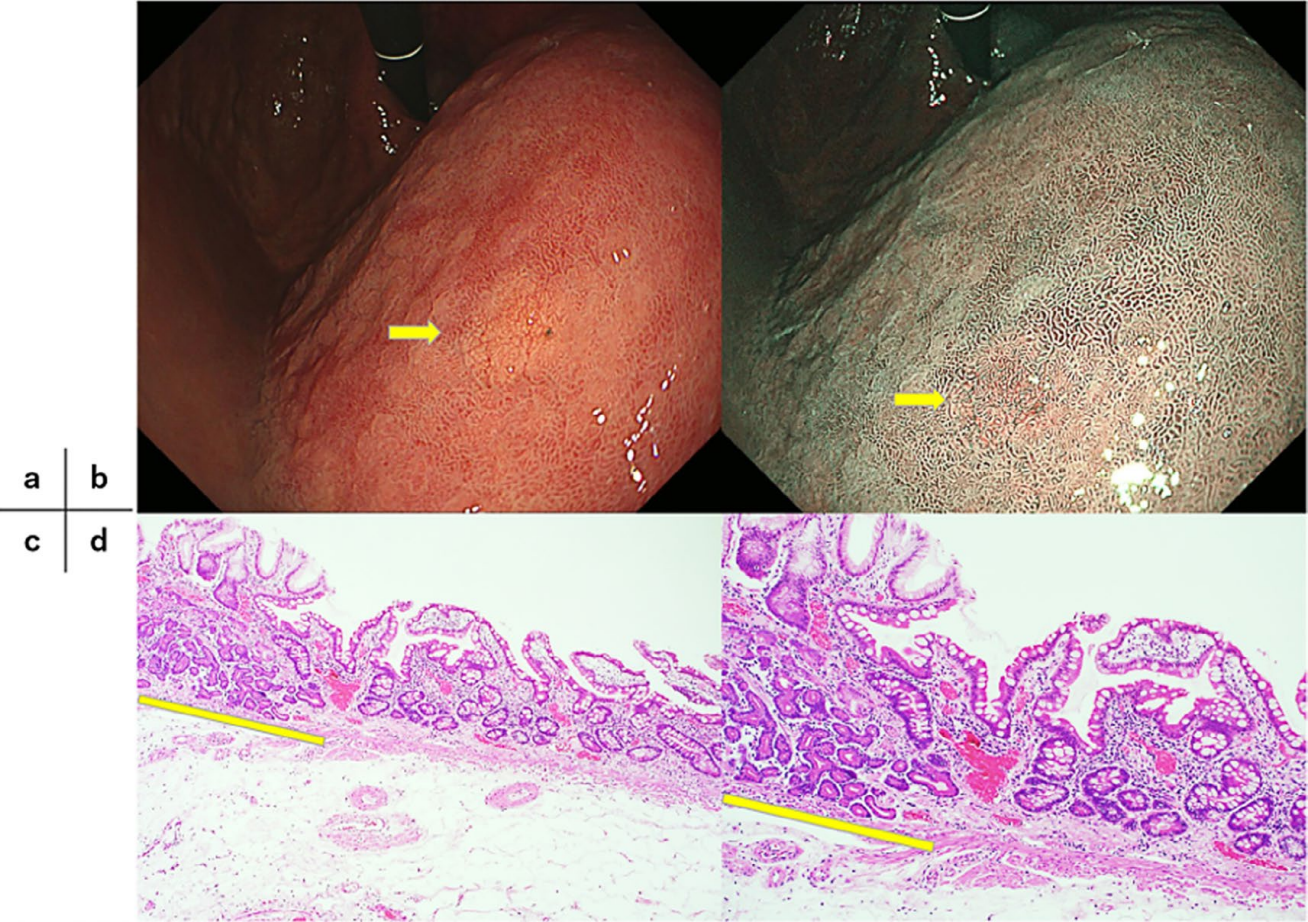
GA‐FG in endoscopically atrophic mucosa, measuring 6 mm in diameter with submucosal invasion of 25 μm (SM1). (a, b) Endoscopic findings of GA‐FG. The background gastric mucosa exhibited atrophic gastritis (O‐3) after *Helicobacter pylori* eradication. The neoplasm was located in the anterior wall of the middle gastric body within the atrophic mucosal area (yellow arrow). (c) Histopathological appearance with Hematoxylin and eosin staining (×40). The neoplasm is indicated with a yellow line. (d) Histopathological appearance with Hematoxylin and eosin staining (×100). Histopathologically, the background gastric mucosa surrounding the neoplasm demonstrated atrophy (USS 3+) and intestinal metaplasia (USS 3+).

**FIGURE 2:**
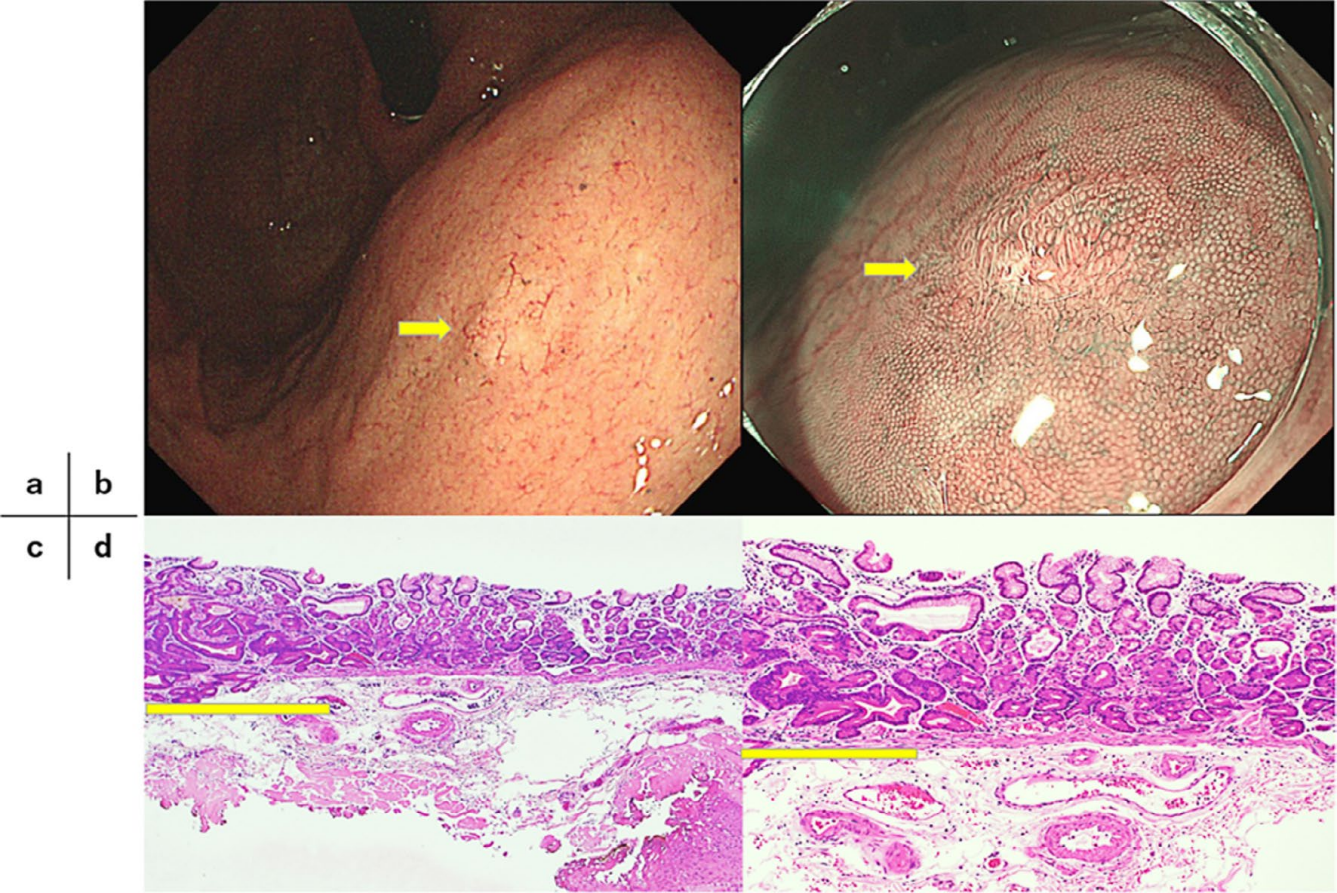
GA‐FG in endoscopically nonatrophic but histologically mildly atrophic mucosa, measuring 5 mm in diameter with submucosal invasion of 130 μm (SM1). (a, b) Endoscopic findings of GA‐FG. The background gastric mucosa showed atrophic gastritis (C‐3) after *Helicobacter pylori* eradication. The neoplasm was located in the anterior wall of the middle gastric body within the endoscopically nonatrophic mucosal area (yellow arrow). (c) Histopathological appearance with Hematoxylin and eosin staining (×40). The neoplasm is indicated with a yellow line. (d) Histopathological appearance with Hematoxylin and eosin staining (×100). Histopathologically, the background gastric mucosa surrounding the neoplasm demonstrated atrophy (USS 1+) without evidence of intestinal metaplasia.

A clinicopathological comparison between the *H. pylori* ‐uninfected and ‐infected groups is shown in Table 2. In the uninfected group, macroscopic types were 0‐IIa/0‐IIb/0‐IIc = 8/2/5, whereas in the infected group they were 5/8/2. The median tumor size was 6 mm (range, 3–10 mm) and 5 mm (range, 2–10 mm) in the uninfected and infected groups, respectively, with both groups primarily showing small lesions. Depth of invasion was T1a (M)/T1b (SM1)/T1b (SM2) = 5/8/2 in the uninfected group and 7/8/0 in the infected group. None of the clinicopathological parameters differed significantly between the groups. Histopathological analysis of background gastric mucosa revealed no atrophy or intestinal metaplasia in any lesion in the uninfected group. However, in the infected group, histological atrophy was observed in 12 of 15 lesions, with USS grades of −/1+/2+/3+ = 3/10/1/1, and intestinal metaplasia was present in three lesions (USS −/1+/2+/3+ = 12/1/1/1). Notably, 13 of 15 lesions (86.7%) in the infected group were endoscopically located in nonatrophic areas. Moreover, mild histological atrophy (USS 1+) was observed in 10 of these lesions. Representative cases are shown in Figures 1 and 2.

**TABLE 2:** Comparison of cases based on *Helicobacter pylori* infection status.

Patients (*n* = 28)	Uninfected (*n* = 13)	Infected (*n* = 15)	*p*
Sex (male/female)	11/2	6/9	0.024
Age (median)	76 [59–81]	76 [61–83]	0.564
Degree of endoscopic gastric mucosal atrophy (C0/C1–C2/C3–O1/O2–O3)	13/0/0/0	0/2/8/5	< 0.01

Lesions (*n* = 30)	Uninfected (*n* = 15)	Infected (*n* = 15)	*p*
Location (U/M/L)	12/3/0	10/5/0	0.682
Macroscopic type (elevated/flat/depressed)	8/2/5	5/8/2	0.071
Color tone (reddish/similar coloration/whitish)	1/3/11	1/1/13	0.791
Size of tumor (mm, median [range])	6 [3–10]	5 [2–10]	0.699
Depth [T1a(M)/T1b(SM1)/T1b(SM2)]	5/8/2	7/8/0	0.477
Depth of invasion (μm, median [range], *n* = 18)	163 [11–1250]	100 [25–462]	0.689
Lymphatic and/or venous invasion (−/+)	14/1	15/0	1
Horizontal and vertical margin (−/+)	15/0	15/0	1
Endoscopic atrophy around the lesion (−/+)	15/0	13/2	0.483
Histopathological atrophy around the lesion (−/+)	15/0	3/12	< 0.01
Histopathological atrophy USS (−/+/++/+++)	15/0/0/0	3/10/1/1	< 0.01
Histopathological intestinal metaplasia around the lesion (−/+)	15/0	12/3	0.224
Histopathological intestinal metaplasia USS (−/+/++/+++)	15/0/0/0	12/1/1/1	0.224

Abbreviation: USS, Updated Sydney System.

## 4 Discussion

In this study, we analyzed the clinicopathological characteristics of 30 lesions in 28 cases diagnosed as OGA or GA‐FG at our institution, focusing on the histopathological features of the background gastric mucosa surrounding the lesions. Cases occurred more frequently in older male patients and were primarily localized in the fundic‐gland region of the upper gastric body. Although the tumor size was small, submucosal invasion was frequently observed, consistent with previous studies [3, 9, 10, 11]. OGA and GA‐FG have traditionally been considered more prevalent in *H. pylori* ‐uninfected stomachs [10, 12, 13]; however, with the accumulation of cases, it has become evident that these tumors can develop regardless of *H. pylori* infection status [14, 15, 16, 17, 18]. In the present study, no lesions were derived from patients with current infection. All cases were *H. pylori* ‐negative, either uninfected or had past infections, with both groups represented in nearly equal numbers.

Ueyama et al. [4] reported no significant differences in clinicopathological features, apart from age and the Ki‐67 MIB1 labeling index, between the *H. pylori* ‐negative group and the *H. pylori* ‐positive or eradicated group. Consistent with these findings, clinicopathological features did not differ significantly between the *H. pylori* ‐uninfected and ‐infected groups in our study.

Previous studies on GEN‐FGML often lacked detailed methods for evaluating gastric mucosal atrophy, and few reported the degree of histopathological atrophy. Notably, Chiba et al. [19] assessed atrophy using the USS visual analogue scale, although their sample size was small. In contrast, we analyzed a larger number of cases, allowing a more comprehensive evaluation. Chiba et al. examined nine cases of OGA or GA‐FG that underwent ESD; seven showed no histopathological atrophy, whereas two exhibited mild and moderate atrophy, one of which had been endoscopically assessed as arising from a nonatrophic mucosal area. In the present study, a comparison based on *H. pylori* infection status revealed histopathological atrophy in 12 of 15 lesions in the infected group. The majority were mild (1+), with only a single lesion each exhibiting moderate (2+) and severe (3+) atrophy. Both lesions with moderate or severe atrophy were located in endoscopically atrophic mucosal areas, whereas the 10 lesions with mild atrophy were found in endoscopically nonatrophic mucosal areas. This underscores the importance of histopathological assessment, as OGA and GA‐FG may arise not only from mildly atrophic mucosa with preserved fundic glands in past‐infected stomachs but also, albeit less frequently, from severely atrophic mucosa. Recognizing that OGA and GA‐FG may develop regardless of background mucosal atrophy could improve lesion detection during routine endoscopic examinations.

Discrepancies between endoscopic and histological atrophy evaluation warrant further discussion. Kono et al. [20] reported high concordance between endoscopic atrophy grading via the Kimura–Takemoto classification [5] and histological atrophy [weighted κ = 0.81 (95% CI: 0.75–0.87)]. However, they also found that extensive histological atrophy was underestimated endoscopically as antrum‐predominant atrophy in 3.6% of cases. The causes of these false‐negative findings were not defined; however, they may have been due to indistinct atrophy borders, uneven atrophy distribution, or interobserver variability. In our study, lesions judged endoscopically as nonatrophic but histologically showing mild atrophy (USS 1+) may belong to this discordant category.

This study has several limitations. First, it was a single‐center retrospective analysis with a relatively small sample size. Second, histopathological evaluation was performed by one pathologist, introducing potential subjectivity. In particular, the USS for assessing atrophy in background gastric mucosa includes subjective elements, and interobserver variability in the histological assessment of gastric atrophy has been well documented [21]. Third, *H. pylori* infection status was not rigorously confirmed using multiple diagnostic methods, possibly affecting classification accuracy. Excluding GA‐FGM represents another limitation of this study, as GA‐FGM differs from OGA and GA‐FG in biological and histogenetic aspects, and its inclusion could have confounded the analysis. Future multicenter collaborative studies are needed to address these limitations.

In conclusion, OGA and GA‐FG frequently arise from nonatrophic mucosa in *H. pylori* ‐uninfected patients and from mildly atrophic mucosa with preserved fundic glands in previously infected patients. Although rarely, they may also develop from severely atrophic mucosa. During routine endoscopic examinations, careful observation of the fundic‐gland area is warranted regardless of the presence of background mucosal atrophy.

## Conflicts of Interest

The authors declare no conflicts of interest.

## Data Availability

The data that support the findings of this study are available on request from the corresponding author. The data are not publicly available due to privacy or ethical restrictions.
